# New York Odontological Society

**Published:** 1885-04

**Authors:** 


### NEW YORK ODONTOLOGICAL SOCIETY.

REGULAR MEETING, HELD AT THE RESIDENCE OF DR. S. G. PERRY, FEBRUARY 17, 1885.

A paper was read by Dr. E. A. Bogue, having for its subject certain articles published in the New York *Medical Record*, on the "Retention of Dead Teeth in the Jaws." He said, first, that *pulpless*, and not dead teeth, are under consideration.

Fillings are not the direct means of retaining pulpless teeth, but are stoppers to prevent the entrance of deleterious matter through the apical foramina, or through cavities opening into the mouth.

Fillings should be inserted before suppuration and the throwing off of mephitic products has begun.

If, through neglect, the pulp has become decomposed, the putrefactive products should be removed, root canals cleansed, deodorized, dried, and asepticized like a mummy. When this is done the contents of the dentinal tubuli are rendered bland and innocuous as a hard boiled egg.

The cementum being of low vascularity, is no more affected by contact with dentine thus treated, than by the vitrified dentine of the octogenarian, and the tooth fulfills its function with comfort.

If a dead pulp be allowed to remain in the tooth, the cementum may be contaminated through the dentinal tubuli, and the pericementum absorb the morbific matter.

Many failures in treatment of teeth with dead pulps may be attributed to a non-recognition of this fact.

Teeth having their pulps removed, root canals made aseptic and accurately filled, even with cotton, seldom give any trouble; but teeth containing dead pulps are open to most of the objections recently brought against them.

Arsenic used as an obtunder has often caused death of pulps, long after the operations have terminated.

Teeth "divested of periostial nourishment by tartar" usually cannot be retained in the mouth, and often fall out of themselves. Tartar may be an effect, and not a cause of disease.

If diseased teeth, which originate the reflex troubles met with by oculists and aurists, cannot be treated so as to render them comfortable, there is no doubt as to the advisability of extraction.

In conclusion, Dr. Bogue said he should be glad to have medical schools give sufficient instruction to enable physicians to diagnose pulpless teeth in a good condition from dead teeth.

MARCH MEETING.

A regular meeting of the Society was held at the residence of Dr. C. E. Francis, 33 W. 47th St., Tuesday evening, March 17th, Dr. W. Jarvie, presiding.

*Dr. S. G. Perry*—Exhibited a plaster cast representing teeth much abraded on their anterior surfaces, and supposed to have been the result of vigorous brushing.

*Dr. C. E. Francis*—Did not believe such conditions should be charged to the use of the tooth-brush. He had seen many cases where teeth were badly abraded, which he was positive were not injured in this manner. He had, that very day, seen in the mouth of a gentleman a number of teeth deeply grooved, front and back, indeed nearly girdled, and with furrows extending almost to the pulp canals. The abraded surfaces were hard and smooth, yet much stained, showing little evidence of over-brushing. He was puzzled to know what could have caused the peculiar condition of these teeth.

*Dr. J. B. Rich*—Described a case where the teeth were seriously denuded, and in such a way that the trouble could not have occurred by brushing. He also cited another case in which an excellent set of teeth became ruined by a too free use of lemons. The enamel was dissolved away, and the dentine so eroded all around that the pulps were nearly exposed. It was the most astonishing destruction of tooth structure he had ever witnessed, and the mischief had all occurred within a brief period, he having examined the teeth about a year before and pronounced them in an excellent condition.

*Dr. F. Y. Clark*—Thought that some agents or exudation from the margin of the gum, had much to do in causing the conditions described.

*Dr. N. W. Kingsley*—Believes that abrasion is due to conditions, and that other agents besides the brush aids in this destruction. He instanced a case where entire surfaces of a number of teeth were abraded, probably by the brush in part, but assisted by other agents. Statements of abrasion from brushing, he thinks, are much exaggerated.

Digressing from the subject, Dr. Kingsley took the occasion to speak of a favorite drill which he had the misfortune to break, but to his delight he found that the broken end of the stub proved an excellent substitute, and would cut splendidly. He suggested the idea of using broken drills, which were sometimes better than the original.

*Prof. Frank Abbott*—Formerly thought that the abrasion of teeth was due more to a solvent than the brush. He had, however, since discovered that natural grooves or waves which are sometimes found on the surfaces of teeth, are starting points for such trouble. The brushes get into these furrows and work them deeper, and solvents assist in the destruction.

*Dr. F. H. Lee*—Presented the root of a central incisor to which was attached a tooth crown of the Buttner style. It was a specimen of "prosthetic dentistry," from a somewhat noted establishment in New York. It had caused much annoyance and pain, and Dr. Lee extracted it. In preparing the root for pivot-anchorage, the drill, instead of following in line with the pulp canal, had diverged, passing diagonally across and through the root into the alveolar territory, and the end of the gold pivot appeared on the side of the root. As a specimen of " high art " it was viewed in the light of a curiosity.

*Dr. W. T. Laroche*—Read a very interesting paper, relating his experience in a case which, viewed from a medical standpoint, is worthy of much consideration. A gentleman, twenty-seven years of age, suffering intense pain of a neuralgic character, called on Dr. Laroche to consult him in regard to his condition. He had already been treated by ten physicians, all of whom made efforts to relieve his suffering, but without avail. The patient at times became delirious, and was treated for brain disorder, but with no benefit. A celebrated specialist was consulted, who, after carefully examining the patient, advised him to call on Dr. Laroche, as he thought his teeth might in some way be the cause of his suffering. In looking carefully into the matter, Dr. L. came to the conclusion that the pain was due to a retarded development of the third molars, which caused irritation to the facial nerves, so he decided on their removal. It was found, however, that their position rendered this a most difficult undertaking; indeed, they could not be reached. As it seemed imperative that relief should be afforded, Dr. L. extracted the twelfth year molar on the side most affected, with the view of making room for the wisdom tooth to work up. The pain was lessened on that side, but on the opposite side was so severe that the other superior second molar was taken out. For a time the patient felt somewhat easier, yet at intervals the pain would recur, and after a time was as violent as ever before, and the patient became nearly frantic. It was then decided that the inferior second molars must be removed, and these in turn were extracted The roots of all the second molars were considerably exostosed.

As with previous operations, a modification of pain for a time followed, but soon to return with unwonted vigor. Searches were subsequently made for the third molars, which in the course of time were taken out, yet with extreme difficulty, as they were so deeply and firmly imbedded in the bone. The roots of these were also exostosed.

With the removal of the last wisdom tooth the pain, which for so long a time had baffled all efforts to dispel, disappeared, and the patient recovered health, strength and comfort. The treatment at the hands of Dr. Laroche covered a period of nearly three years.

The whole trouble was evidently due to exostosis, which previous medical treatment failed to control, and was only alleviated by a well-directed use of forceps.

